# Soil organic carbon dynamics under long-term fertilization in a black soil of China: Evidence from stable C isotopes

**DOI:** 10.1038/srep21488

**Published:** 2016-02-22

**Authors:** Xiaolin Dou, Ping He, Ping Zhu, Wei Zhou

**Affiliations:** 1 Institute of Agricultural Resources and Regional Planning, Chinese Academy of Agricultural Sciences (CAAS), Beijing 100081, P. R. China; 2 Jilin Academy of Agricultural Sciences, Gongzhuling 130124, P. R. China

## Abstract

Effects of different fertilizers on organic carbon (C) storage and turnover of soil fractions remains unclear. We combined soil fractionation with isotope analyses to examine soil organic carbon (SOC) dynamics after 25 years of fertilization. Five types of soil samples including the initial level (CK) and four fertilization treatments (inorganic nitrogen fertilizer, N; balanced inorganic fertilizer, NPK; inorganic fertilizer plus farmyard manure, MNPK; inorganic fertilizer plus corn straw residue, SNPK) were separated into four aggregate sizes (>2000 μm, 2000–250 μm, 250–53 μm, and <53 μm), and three density fractions: free light fraction (LF), intra-aggregate particulate organic matter (iPOM), and mineral-associated organic matter (mSOM). Physical fractionation showed the iPOM fraction of aggregates dominated C storage, averaging 76.87% of SOC storage. Overall, application of N and NPK fertilizers cannot significantly increase the SOC storage but enhanced C in mSOM of aggregates, whereas MNPK fertilizer resulted in the greatest amount of SOC storage (about 5221.5 g C m^2^) because of the enhanced SOC in LF, iPOM and mSOM of each aggregate. The SNPK fertilizer increased SOC storage in >250 μm aggregates but reduced SOC storage in <250 μm aggregates due to SOC changes in LF and iPOM.

Agro-ecosystem represents around 40% of all land on earth^1^, which is critical for maintaining agricultural sustainability, environmental stability, and long-term terrestrial carbon (C) sequestration^2^,^3^. Soil organic carbon (SOC) plays an important role in cycling plant nutrients, increasing grain yield and improving the physical, chemical properties of soils^4^. Fertilization as an agricultural management strategy, is being used to promote soil C storage^5^,^6^, which could directly or indirectly increase the SOC inputs and thereby change the availability of nutrients and soil turnover^1^. For instance, inorganic nitrogen (N) fertilizer may indirectly enhance the SOC storage by increased crop residue input to soils^5^,^7^, whereas application of organic manure could influence soil organic matter (SOM) owing to the directly inputs of processed organic materials to soils^8^,^9^,^10^,^11^. In contrast, application of organic fertilizers would result in a higher level of soil C and N mineralization than inorganic fertilizers^9^,^10^,^12^.

Some studies have shown that inorganic fertilizers had important effects on crop yield but no significant effect on SOM or SOC^8^,^12^,^13^. However, the combined application of inorganic and organic fertilizers has been shown to improve SOM better than the simple addition of inorganic fertilizer^8^,^12^,^14^. To date, positive^5^,^15^,^16^, negative^4^, and no obvious effects^9^,^12^,^13^ have been reported under fertilization on soil C sequestration for agro-ecosystems. The above controversy may be explained by the fact that increased SOM input resulting from fertilizers may be offset by the soil C loss from various soil fractions, resulting in zero accumulation of SOC, even a negative deficit^8^,^17^. Therefore, insight is urgently needed into soil C dynamics under long-term fertilization.

Generally, soil C turnover mainly depends on the interplay between organic inputs (e.g. plant debris, organic fertilizer) and decomposition of SOM with positive, neutral, or even negative response to fertilizer additions^18^. Thus, understanding the changes in new soil C inputs and the decay rate of old C will be essential to revealing soil C dynamics under long-term fertilization. Originally, the source and turnover rates of SOC can be assessed since δ^13^C values of C_3_ (δ^13^C *ca*. −28‰) and C_4_ (δ^13^C *ca*. −12‰) vegetation are distinctly different because of their differences in utilizing C isotopes^2^,^19^. The relative contribution of new SOC *vs*. old SOC can be estimated based on the mass balance of C isotope contents, and thus SOM turnover rate could then be estimated *in situ*^19^,^20^,^21^. In the present study, soil C turnover was quantified using δ^13^C abundance based on the changes in decomposition level after 25 years of fertilization^6^,^22^. Additionally, detecting changes in soil C dynamics can be difficult, for the SOM consists of a variety of compounds with different microbial degradability and turnover time^23^. For instance, macroaggregation formed around fresh coarse residues was more sensitive to agricultural practices than microaggregation^24^. Meanwhile, the light fraction commonly referred to a plant-like and less stable fraction due to contain physically unprotected plant debris^25^, whereas the heavy fraction was shown to be a major sink for C storage with a more stable fraction due to more recalcitrant component^26^. Thus, the SOM physical fractionation technique together with natural abundance in stable C isotopic composition, has been considered to be an effective approach for quantifying SOM dynamics under long-term fertilization in agro-ecosystems^1^,^6^,^11^.

Black soils (Mollisols) with a rich organic matter content, are the most fertile and productive soils in Northeast China^27^. In recent years, the productivity of the black soils has been declining as a result of unsustainable agricultural practices^10^. In the agricultural tillage system of China, aboveground crop residue is usually removed for energy use or as livestock feed, which could result in a decline of SOM, a depletion of C stocks, deterioration of soil structure, and serious soil erosion. Therefore, various fertilizers (e.g., N fertilizer and manure) are applied in cropland to improve the SOM quality and quantity and to help increase the crop yield^27^. In this study, we hypothesized 25 years of fertilization would significantly change organic C storage of soil fractions and turnover rate of soil C (the proportion of soil new *vs*. old C). The objectives of this study were to examine the following issues: (1) how long-term fertilization has potentially impacted the organic C storage in the SOM fractions; and (2) how long-term fertilization affects the new C inputs and decay rates of old C in the native SOM fractions.

## Results

### The soil physicochemical properties and plant biological traits

The soil total C and N, SOC content was greater in MNPK- and SNPK-treated soils and lower in N- and NPK-treated soils than in the initial level (CK) (Table 1). The soil bulk density was significantly higher in N- and NPK-treated soils than in MNPK- and SNPK-treated soils and CK, whereas soil pH had an opposite tendency with the lowest pH values (pH = 6.3 and 6.4) in N- and NPK-treated soils (Table 1). The δ^13^C values of the leaf and roots varied from −13.36‰ to −16.23‰ and from −12.67‰ to −14.35‰, respectively, in the corn-planted field, which was typical of C_4_ plants (Table 1). The C:N ratios in the leaf and roots of the corn decreased in the following order: SNPK >MNPK >NPK >N-treated soils (Table 1).

### Size distribution, SOC storage, and δ^13^C of soil aggregates

**Figure 1:**
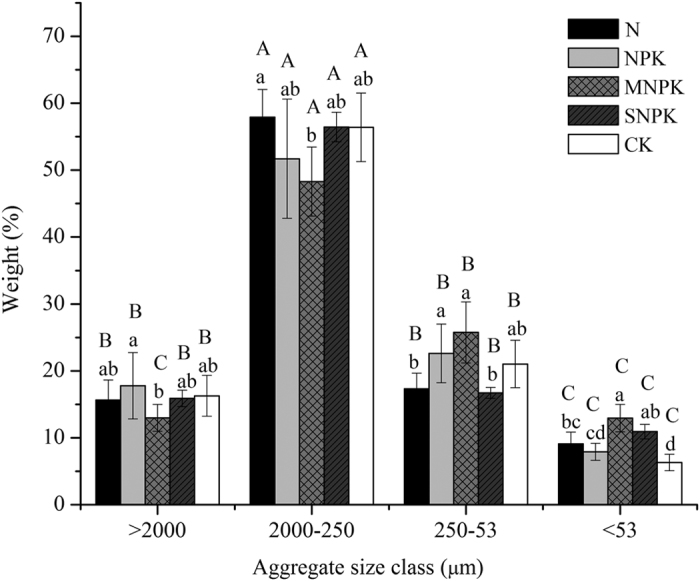
Portion of various aggregate size classes separated from soils (0–20 cm) under long-term fertilization. Values followed by a different lowercase letter over the bars of root indicate statistically significant differences at *P* < 0.05 within aggregate size classes among fertilization treatments. Values followed by a different capital letter over the bars of root indicate statistically significant differences at *P* < 0.05 among aggregate size classes under different fertilization treatments.

Long-term fertilization cannot significantly affect the portioning of aggregate distribution across the fertilizer treatments except for <53 μm aggregates (Fig. 1). Overall, application of organic and inorganic fertilizers increased the weight distribution of <53 μm size fraction compared with CK (Fig. 1). In general, the aggregate distribution was dominated by macroaggregates (2000–250 μm; 48.31–64.10%) across all the fertilizer treatments.

**Figure 2:**
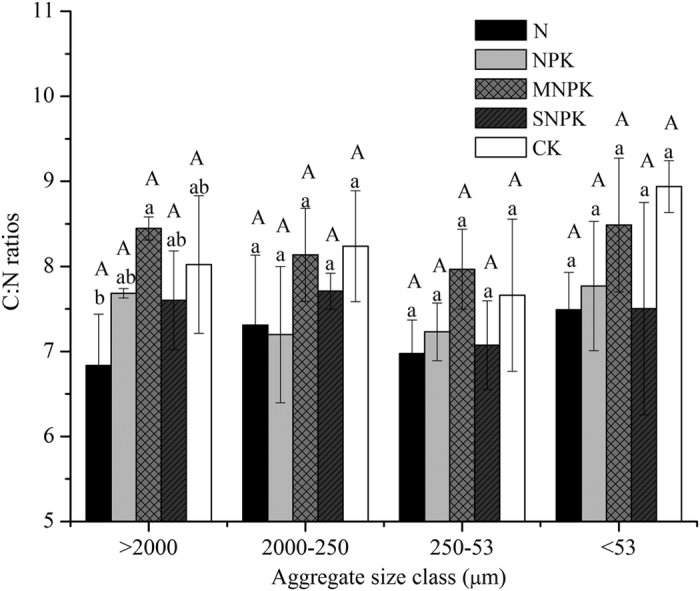
C: N ratios of soil aggregate size classes separated from soils (0–20 cm) under long-term fertilization. See Fig. 1 for the explanation of the different letters.

Overall, long-term application of N and NPK fertilizers resulted in no remarkable increases in SOC storage across all aggregates compared with CK, except for 2000–250 μm aggregates in N-treated soils (Table 2). Long-term MNPK fertilizer strongly increased the SOC storage by an average of 466.0 g C m^2^ in all aggregates. The SNPK fertilizer increased SOC by an average of 191.1 g C m^2^ in macroaggregates (>250 μm) but decreased it by an average of 131.4 g C m^2^ in microaggregates (250–53 μm) compared with CK (Table 2). Besides, the SOC storage showed a decrease in 250–53 μm aggregates compared with other aggregate sizes in the fertilized soils except for MNPK treatment. Generally, the SOC storage in macroaggregates (>250 μm) was greater than in microaggregates (<250 μm) across the fertilizer treatments (Table 2). Long-term fertilization resulted in no significant changes in the C:N ratios across all the aggregate sizes (Fig. 2). The δ^13^C values of all of the aggregates were less negative in the fertilized soils than in CK due to the C_4_ residue inputs, whereas the least negative δ^13^C values appeared in SNPK-treated soils (Table 3). Overall, the least negative δ^13^C values were found in 250–53 μm aggregates across all the aggregate sizes (Table 3).

### Density fractionation: SOC storage and δ^13^C in LF, iPOM and mSOM

**Figure 3:**
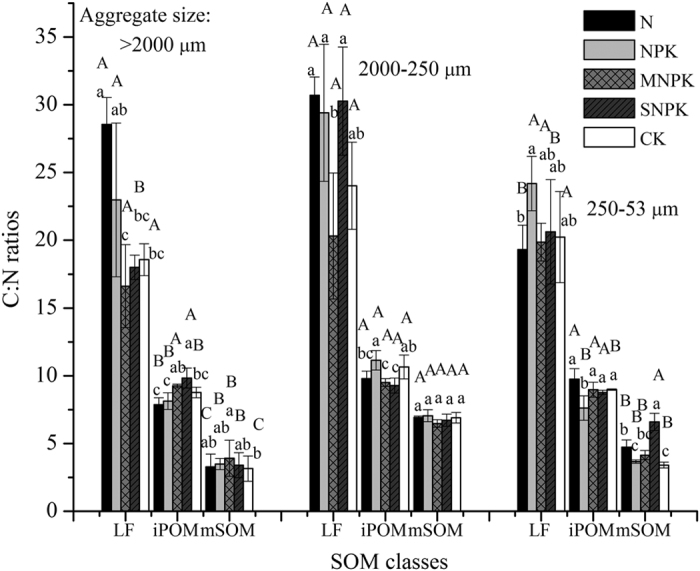
C: N ratios of LF, iPOM and mSOM of aggregate size classes separated from the soils (0–20 cm) under long-term fertilization. Values followed by a different lowercase letter over the bars of root indicate statistically significant differences at *P* < 0.05 within SOM classes among fertilization treatments of each aggregate size. Values followed by a different capital letter over the bars of root indicate statistically significant differences at *P* < 0.05 among SOM classes under different fertilization treatments of each aggregate size.

The iPOM stored the largest C fraction of the total SOC pool across all the aggregate sizes in the fertilized soils, which accounted for 80.79–90.32% in >2000 μm and 250–53 μm aggregates, and as well as accounted for 49.59–63.89% in 2000–250 μm aggregates (Table 2). The mSOM accounted for the smallest fraction (1.54–4.92%) of total organic C in >2000 μm aggregates, whereas the LF accounted for the smallest fraction (4.10–6.74%) of total organic C in 250–53 μm aggregates (Table 2). Particularly, the largest SOC storage in mSOM appeared in 2000–250 μm aggregates (Table 2). The greatest SOC storage in the LF, iPOM, and mSOM of all the aggregates was found in MNPK-treated soils (Table 2). The SNPK fertilizer obviously enhanced SOC storage mainly in LF and iPOM of macroaggregates (>250 μm) but decreased SOC in LF and iPOM of microaggregates (<250 μm) (Table 2). Additionally, inorganic N and NPK fertilizers significantly increased the SOC storage in mSOM of all the aggregates. Moreover, inorganic fertilizers increased the LF in >2000 μm aggregates but decreased it in 250–53 μm aggregates (Table 2). No significant changes of SOC storage in iPOM were found in N- and NPK-treated soils compared with CK across all aggregate sizes (Table 2). The higher C:N ratios occurred in LF while the lower C:N ratios occurred in mSOM among soil density fractions across all fertilization treatments (Fig. 3). Long-term fertilization led to less negative δ^13^C values in LF, iPOM, and mSOM compared with CK, and the least negative δ^13^C values were found in SNPK-treated soil across all the soil fractions (Table 3). Furthermore, the most negative δ^13^C values appeared in LF in the fertilized soils among soil density fractions of each aggregate size, while the least negative δ^13^C values appeared in mSOM of the macroaggregates (>250 μm) (Table 3).

### Soil C turnover

Long-term fertilization stimulated both new C inputs and decay rate of old C in soil fractions (Table 4). The new C inputs into all of the aggregates were greatest in SNPK-treated soils followed by MNPK-treated soils (Table 4). Accordingly, the fastest decay rates of old C were found in SNPK-treated soils across all aggregate sizes (Table 4). Overall, the greatest new C inputs into the soil aggregates and the fastest C decay rates were found in 250–53 μm aggregates (Table 4). The new C inputs were greater in mSOM than LF and iPOM in macroaggregates (>250 μm), whereas the fastest soil C turnover occurred in mSOM of 2000–250 μm aggregates (Table 4).

## Discussion

Our stable isotope analysis confirmed that the abundance of δ^13^C in SOM fractions in the fertilized soils was more enriched than in CK (Table 3), resulting from a higher contribution of C_4_ residues^19^,^23^. Overall, we found that the greatest SOC storage was found in MNPK-treated soils, followed by SNPK and then by inorganic fertilizers across all the aggregates (Table 2), which fully supported our previous study and others^9^,^28^,^29^. Furthermore, the SOC storage in macroaggregates (>250 μm) was greater than in microaggregates (<250 μm) overall across the fertilization treatments (Table 2), suggesting that the presence of macroaggregates (>250 μm) is usually associated positively with SOC content as an important component for C sequestration in soils^30^. In our study, the aggregate distribution was dominated by macroaggregates (2000–250 μm; 48.31–64.10%); meanwhile, the iPOM accounted for the largest C fraction (76.87% on average) of the total SOC pool across all the treatments (Table 2). Thus, we may draw the conclusion that 25 years of fertilization significantly increased the SOC storage, mainly by enhancing the soil C of the macroaggregates (2000–250 μm) with most of the C and N stored in the iPOM in the black soils of northeast China.

The SOC storage showed a decrease in 250–53 μm aggregates in the fertilized soils except for MNPK treatment, maybe due to the fastest decay rates of old C in 250–53 μm fractions among aggregate sizes^17^ (Tables 2 and 4). Moreover, the SOC storage in LF with a less stable fraction was susceptible to be decomposed by microorganisms, and indeed maintained the same trend as that in 250–53 μm aggregates (Table 2). The findings suggested that despite the better physically protection against soil C decomposition in microaggregates, SOC within microaggregates may be susceptible to microbial breakdown^6^. Additionally, higher C:N ratios of LF reflected more recent litter inputs, while mSOM with much lower C:N ratios suggested decreasing C:N ratios in soil C fractions have been associated with increasing SOM decomposition and mineral association^23^,^24^ (Fig. 3). Indeed, the decay rates of old C were relatively fast in the mSOM across all the fractions in our study (Table 4). Overall, the C decay rates were relatively slow in N- and NPK-treated soils than in MNPK- and SNPK-treated soils (Table 4), indicating that application of organic fertilizers combined with inorganic fertilizers would accelerate the soil C turnover rate when compared with the addition of inorganic fertilizers alone^4^,^29^.

Overall, we found that there were no significant changes in SOC storage across all the aggregates relative to CK except for in the 2000–250 μm aggregates in N-treated soils after long-term application of N and NPK fertilizers (Table 2), which indicated that long-term N and NPK fertilizers decreased the SOC content, but significantly increased soil density in the 0–20 cm layer^31^ (Table 1). This result confirmed the previous studies that 25 years of continuous inorganic fertilization was not capable of increasing the total SOC relative to the control^8^,^10^. Previous study showed this occurred for the two reasons as stated below^29^. First, inorganic N and NPK fertilizers were insufficient for preserving SOC levels under conventional management due to no above-ground crop residues returning to soil, although inorganic fertilizers may indirectly enhance SOM by increasing plant biomass production and C return to soils^10^. Second, the simple addition of inorganic N and NPK fertilizers lead to the soil acidification^13^, which correspondingly affected soil microbial activity, microbial biomass C and thus affected the SOC pool^32^ (Table 1). Generally, mSOM was shown to be a major sink for C storage with a more stable fraction because of the presence of a more recalcitrant component^26^. Soil density fractionation revealed that soil C storage was greater in mSOM fractions of each aggregate in N- and NPK-treated soils than in CK (Table 2), suggesting that soil inorganic N input may stabilize soil C in heavier fraction to a certain extent^33^,^34^. Additionally, we found that inorganic N and NPK fertilizers cannot increase the SOC storage in microaggregates (<250 μm), probably because of the offsetting effects of enhanced the mSOM and decreased the LF in microaggregates. The above findings further supported the previous analysis, which showed that no apparent changes in SOC storage of total organic pools occurred in N- and NPK-treated soils, mainly owing to the offsetting effects between enhanced SOC in the recalcitrant pool and decreased SOC in the labile pool^29^.

In contrast, a long-term application of MNPK fertilizer resulted in the largest soil C storage (about 5221.5 g C m^2^) among fertilization treatments, which strongly increased the SOC storage on average by 1863.9g C m^2^ at the black soil region of northeast China (Table 2), which further supported the evidence that long-term addition of manure significantly increased SOC content, regardless of combining inorganic fertilizers or not^8^. The SOC storage is the net effect of organic matter inputs to soil and losses through decomposition^35^. In our study, increased SOC storage in MNPK-treated soils was mainly caused by C accumulation in soils via manure inputs given a high SOC content about 112 g kg^−1^ at the experimental site^10^. Meanwhile, relatively fast decay rates of old C were found in MNPK-treated soils across all the aggregates in our study (Table 4). Thus, our results suggested that the positive effect of manure amendments with a high SOC content was not offset by the fast decay rate of C in MNPK-treated soils^4^,^36^. Additionally, Xie *et al*. (2014)^10^ showed that manure amendments improved the labile SOM pool at the same site^10^. Whereas our previous analysis by chemical fractionation showed the enhanced organic C pool in MNPK-treated soils was related to the increased SOC in both recalcitrant and labile pools^29^. In present study, soil density fractionation further showed that the positive effect of organic C in response to the MNPK fertilizer was ascribed to the increased SOC in all density fractions (LF, iPOM, and mSOM) of each aggregate (Table 2). Thus, we can conclude that long-term application of MNPK fertilizer may be better for future soil C sequestration compared with other fertilizers. Additionally, the LF of SOM, as an early and sensitive indicator of the response to the long-term effects of agricultural practices^25^, indicated that improvement of SOM in MNPK-treated soils may be first ascribed to a decline of C/N ratios in LF^8^ (Fig. 3).

The previous studies showed that short-term (e.g. 2–4 years) straw return treatment combined with inorganic fertilizer addition was beneficial for the accumulation of SOC and labile organic C content compared with the no straw addition treatment at the top soil^37^,^38^. Our present results further revealed long-term SNPK fertilization eventually increased SOC storage by an average of 191.1 g C m^2^ in macroaggregates (>250 μm) compared with CK, mainly because of the enhanced organic C in LF and iPOM, while it reduced SOC storage in microaggregates (<250 μm), mainly due to the decreased C in LF and iPOM (Table 2). Straw was a low-quality organic resource with a high C:N ratio, and thus has a slow decomposition rate^36^,^39^. In fact, the fastest decay rates of old C were found in SNPK-treated soils across all the aggregates (Table 4), which was inconsistent with previous studies that slow aggregate turnover had been observed with low-quality organic resources^40^. However, the above findings fully coincided with our previous study that corn straw combined with inorganic fertilizers could accelerate the soil C turnover, and thus result in a larger new C input and faster decay rate of old C compared with the simple addition of inorganic fertilizers or straw alone^29^. This is because that straw decomposed slowly, but the addition of N fertilizers could negate some effects of this type of low-quality organic resource^39^. Our present physically fractionation further confirmed that corn straw combined with inorganic fertilizers (SNPK fertilizer) could accelerate the soil C turnover, largely through various sizes of soil aggregates including macroaggregates and microaggregates (Table 4).

To conclude, we build on our previous findings and utilize the natural abundance of δ^13^C together with soil physical fractionation technique to evaluate dynamics in the SOC fractions after 25 years of fertilization. The present results further confirmed the previous study conducted by soil chemical fractionation technique^29^ and suggested that long-term application of fertilization could alter the SOC storage, consequently affecting the dynamics of soil C pools in agro-ecosystem. These findings will be helpful for monitoring long-term C-accumulation through ecosystem processes under agricultural management practices in a black soil of Northeast China.

## Materials and Methods

### Study area

A long-term fertilization experiment presented for monitoring black soil fertility and fertilizer efficiency with monoculture maize (*Zea mays L*.) has been conducted since 1989 at Gongzhuling, Jilin Province, China (124°48′33″E, 43°30′23″N)^10^,^31^. This region has a north temperate and semi-humid climate with an annual average temperature of 5.6 °C. The annual precipitation is approximately 562 mm, 80% of which falls between June and September^31^. The soil is a clay loam [Typic Hapludoll (Mollisol) in USDA Soil Taxonomy] developed from Quaternary loess-like sediments with 39% sand, 30% silt and 31% clay at the beginning of the experiment^10^. A randomized complete block design was used with three replicates in this long-term experiment with each replicate plot covered 130 m^2^. The experiment included five treatments: (1) Initial soils (CK); (2) inorganic nitrogen fertilizer at the rate of 165 kg N ha^−1^ (N); (3) balanced inorganic fertilizers at 165 kg N ha^−1^, 82.5 kg P_2_O_5_ ha^−1^, and 82.5 kg K_2_O ha^−1^ (NPK); (4) balanced inorganic fertilizers at 50 kg N ha^−1^, 82.5 kg P_2_O_5_ ha^−1^, and 82.5 kg K_2_O ha^−1^ plus farmyard manure at the rate of 2.3 × 10^4 ^kg ha^−1^ (MNPK), and (5) balanced inorganic fertilizers at 112 kg N ha^−1^, 82.5 kg P_2_O_5_ ha^−1^, and 82.5 K_2_O kg ha^−1^ plus corn straw residue at the rate of 7.5 × 10^3^ kg ha^−1^ (SNPK)^31^,^41^. The N contents in corn straw and farmyard manure were 7.0 and 5.0 g kg^−1^, respectively, and thus the total N application rates for N, NPK, SNPK, and MNPK treatments were kept at 165 kg ha^−1^ (dry weight basis)^31^. The organic C content of farmyard manure (mostly, pig manure) was about 112 g kg^−1^^10^; the δ^13^C of farmyard manure was measured with an average value of −21.59‰. The sources of inorganic N, P, and K fertilizers were urea, triple superphosphate (TSP) and muriate of potash (MoP)^31^. One third of the urea and total amounts of TSP and MoP were applied as a basal dose. The application of fertilizers was approximately 10 cm of soil depth. The remaining two thirds of the urea was used for side dressing at the corn jointing stage, whereas the chopped corn straw was also applied in the SNPK plots with the top 25 cm of soil at that time every year. The farmyard manure was applied in the MNPK plots after corn harvesting in autumn each year. Corn was sown in late April and harvested in late September. Aboveground plant residues were removed at harvest^31^. Prior to the long-term experiment, the field had been continuously cultivated corn for some years, and then was homogenized by growing corn for 3 years without fertilizer application^10^. The soil physiochemical properties (pH, bulk density, C and N content) were shown in Table 1. The pH and bulk density of soil were measured as previously described by Song *et al*. (2015)^31^.

### Field sample collection and soil fractionations

In August 2014, we randomly placed three sub-plots (2 m × 2 m) around the corn rhizosphere within each treatment plot; the distances between the sub-plots were approximately 5 m. Soil samples (0–20 cm) from each treatment plot were collected using a 5-cm diameter stainless steel soil corer. Newly produced corn leaves were collected in each treatment plot. Root sampling blocks were excavated within a 30 × 30 cm quadrant at a soil depth of 0–20 cm and then were washed clean carefully; leaves and roots were oven dried to a constant weight at 65 °C in the laboratory to prepare for determination. The soil samples were air-dried, after which the large roots and stones were removed by hand.

The methods for aggregate separation and size density fractionations were adapted from Six *et al*. (1998)^42^. Four aggregate sizes were separated using wet-sieving through a series of sieves (2000, 250, and 53 μm). A 100 g air-dried sample was submerged for 5 min at room temperature (about 25 °C) in de-ionized water on top of the 2000-μm sieve. Aggregate separation was achieved by manually moving the sieve up and down 3 cm with 50 repetitions over a period of 2 min. After the 2-min cycle, the stable >2000 μm aggregates were gently back-washed off the sieve into an aluminum pan. The floating organic material (>2000 μm) was discarded, because this is by definition not considered SOM^42^. The water and soil that passed through the sieve were poured into the next two sieves (one at a time), and the sieving was repeated in a similar procedure; however, floating material was retained. Therefore, four size fractions were obtained (>2000 μm, 2000–250 μm, 250–53 μm and <53 μm). The aggregates were oven dried at 50 °C, weighed, and stored in glass jars at room temperature (about 25 °C).

The density fractionation was carried out by using a solution of 1.85 g cm^−3^ sodium polytungstate (SPT), following the methods described in Six *et al*. (1998)^42^. A subsample (5 g) of each oven-dried (110 °C) aggregate size fraction was suspended in 35 ml of SPT and was slowly shaken by hand. The material remaining on the cap and sides of the centrifuge tube were washed into the suspension with 10 ml of SPT. After 20 min of vacuum (138 kPa), the samples were centrifuged (1250 g) at 20 °C for 60 min. The floating material (light fraction-LF) was aspirated onto a 20 μm nylon filter, washed multiple times with deionized water to remove the SPT and dried at 50 °C. The heavy fraction (HF) was rinsed twice with 50 ml of deionized water and dispersed in 0.5% sodium hexametaphosphate by shaking for 18 h on a reciprocal shaker. The dispersed heavy fraction was then passed through a 53-μm sieve and the remaining material on the sieve, i.e., the intra-aggregate particulate organic matter (iPOM) while the fraction filtered down, i.e., the mineral-associated organic matter (mSOM), was dried (50 °C) and weighted.

### C content and C isotope analyses

The above oven-dried plant materials and collected soil samples were ground to pass through 20-mesh (0.84 mm) sieves. Subsamples from all soil fractions were treated with 1N HCl for 24 h at room temperature to remove any soil carbonates^23^. The C and N content of plant materials (leaves and roots), the whole soil and soil fractions were measured. The δ^13^C values were measured for all soil fractions, plant materials and farmyard manure. Subsamples of leaf, root, and soil fractions were weighed and analyzed using an isotope ratio mass spectrometer (Thermo Finnigen, Delta-Plus, Flash, EA, 1112 Series, USA). The carbon isotope ratio of the soil fractions and plant materials was expressed as follows:


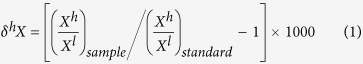


where *X* is carbon, *h* is the heavier C isotope, and *l* is the lighter C isotope. The CO_2_ samples were analyzed relative to the internal working gas standards. The carbon isotope ratios (^13^C) are expressed as relative values to the Pee Dee Belemnite (δ^13^C = 0.0112372‰). The standards (acetanilide and spinach) were analyzed after every ten samples; the analytical precision of the instrument was ±0.13‰ for δ^13^C.

With respect to the plots of different fertilization treatments, the δ^13^C values of the SOM were used to calculate the proportion of new C (*f*_*new*_, i.e. the C derived from current corn residuals or fertilizers) and of old C (*f*_*old*_ = 1−*f*_*new*_, soil C previous to fertilization, i.e., C in the initial soil) with a mass balance equation^43^:


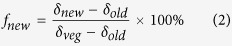


where *δ*_*new*_ is the δ^13^C values of organic C in soil fractions under fertilization, *δ*_*old*_ is the δ^13^C values of organic C from initial soils, i.e. the soil samples previous to fertilization, and *δ*_*veg*_ is the δ^13^C values of the mixed plant materials of corn; Specially, *δ*_*veg*_ is the δ^13^C values of the mixed sample including plant materials and manure in MNPK fertilizer treatment^19^,^22^.

Because the *δ*_*veg*_, *δ*_*new*_ and *δ*_*old*_ are independently measured, the standard errors (SE) of *f* associated with the use of the mass-balance approach can be calculated using partial derivatives^44^ as follows:


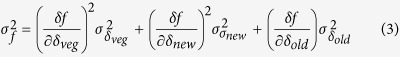


This equation can be reduced to:


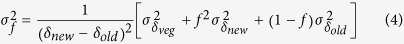


where 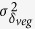, 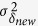 and 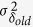 represent the variances of the mean *δ*_*veg*_, *δ*_*new*_ and *δ*_*old*_, respectively. The 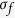 is the SE of the proportion (*f*) estimate^44^.

The decay rate constant (*k*) for the old C (i.e. the C of the organic matter before fertilization) of the soil fractions was calculated based on Cheng *et al*. (2011)^23^:


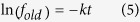


where *f*_*old*_ = (1 – *f*_*new*_) is the proportion of old C, *k* is the net relative decay rate constant for old C, and *t* is the age of fertilization (i.e. for 25 years).

### Statistics

The SOC content, C:N ratios, δ^13^C values, the new C input (*f*_*new*_), and the decay rate (*k*) of the old C of the soil fractions for each treatment were calculated by averaging the three replicates for each sample plot. All of the data were examined for the normality by Kolmogorov–Smirnov test and for the homogeneity of variance by one-way analysis of variance (ANOVA). An analysis of variance (ANOVA) of multiple comparisons was conducted to examine the effects of various fertilization treatments on the on total C and N, bulk density and pH of the whole soil, and the SOC content, the δ^13^C values, the C:N ratios in all soil fractions, and the weight distribution (LSD; *P* = 0.05). One-way ANOVAs were performed to examine the differences in SOC content, δ^13^C value, weight distribution of the soil fractions, new C input (*f*_*new*_), and the decay rate of the old C among fertilization treatments (LSD; *P* = 0.05). All of the statistical analyses were performed using SPSS (version 16.0) and OriginPro (version 8.0) for Windows.

## Additional Information

**How to cite this article**: Dou, X. *et al*. Soil organic carbon dynamics under long-term fertilization in a black soil of China: Evidence from stable C isotopes. *Sci. Rep*. **6**, 21488; doi: 10.1038/srep21488 (2016).

## Figures and Tables

**Table 1:** Soil physiochemical properties (0–20 cm) and plant biological traits under long-term fertilization in Gongzhuling, Jilin Province, China.

Treatments	Soil					*δ*^13^C (‰) – Corn		C:N ratio – Corn	
	TC (g kg^−1^)	TN (g kg^−1^)	SOC (g kg^−1^)	BD (g cm^−3^)	pH	Leaf	Roots	Leaf	Roots
N	15.87 ± 0.72^bc^	1.85 ± 0.06^b^	14.49 ± 1.25^c^	1.39^a^	6.3^b^	−14.84 ± 0.23^a^	−13.35 ± 0.17^ab^	15.39 ± 2.13^c^	24.44 ± 3.67^b^
NPK	14.59 ± 1.53^c^	1.84 ± 0.04^b^	14.16 ± 1.69^c^	1.34^a^	6.4^b^	−13.36 ± 0.25^a^	−14.21 ± 0.21^b^	16.17 ± 0.86^bc^	28.33 ± 2.19^ab^
MNPK	23.47 ± 2.64^a^	2.87 ± 0.34^a^	22.40 ± 1.17^a^	1.21^b^	7.4^a^	−16.23 ± 0.34^b^	−14.35 ± 1.16^b^	16.95 ± 1.43^b^	28.45 ± 4.09^a^
SNPK	17.04 ± 0.28^b^	2.15 ± 0.09^b^	16.79 ± 1.06^b^	1.20^b^	7.7^a^	−14.76 ± 0.56^a^	−12.67 ± 0.33^a^	19.09 ± 3.08^a^	29.56 ± 2.01^a^
CK	16.50 ± 1.23^b^	2.11 ± 0.27^b^	16.14 ± 0.60^b^	1.17^b^	7.6^a^				

Data are expressed as mean ± SE, n = 3. Different letters indicate statistical significance at *P* < 0.05 among fertilization treatments. *Abbreviations*: TC, total carbon; TN, total nitrogen; SOC, soil organic carbon; BD, bulk density; N, inorganic N fertilizer; NPK, balanced inorganic fertilizers of N, P and K; SNPK, balanced inorganic fertilizers plus corn straw residue; MNPK, balanced inorganic fertilizers plus farmyard manure; CK, initial soil.

**Table 2:** Soil organic C storage of soil fractions (0–20 cm) under long-term fertilization.

Fractions	N	NPK	C (g m^−2^)	SNPK	CK
			MNPK		
>2000 μm	600.76 ± 15.78^bc^	596.75 ± 20.19^bc^	710.93 ± 63.20^a^	624.41 ± 34.65^b^	561.17 ± 60.61^c^
LF	75.99 ± 10.04^a^	69.14 ± 8.66^a^	70.35 ± 10.65^a^	52.42 ± 7.21^b^	32.49 ± 5.74^c^
iPOM	514.27 ± 16.59^b^	498.76 ± 52.33^b^	568.88 ± 87.79^a^	557.15 ± 92.05^a^	486.99 ± 68.23^b^
mSOM	10.05 ± 1.78^b^	14.63 ± 3.21^b^	33.07 ± 2.77^a^	9.51 ± 1.24^bc^	6.82 ± 1.06^c^
2000–250 μm	2079.16 ± 308.61^b^	1794.36 ± 221.40^c^	2666.03 ± 352.41^a^	2096.59 ± 104.75^b^	1777.69 ± 250.48^c^
LF	262.84 ± 45.29^b^	262.44 ± 43.69^b^	337.13 ± 54.70^a^	232.31 ± 22.40^b^	252.35 ± 16.92^b^
iPOM	1260.48 ± 273.92^bc^	881.63 ± 94.24^c^	1647.92 ± 166.54^a^	1302.45 ± 156.64^b^	935.32 ± 9.09^c^
mSOM	613.52 ± 43.90^ab^	633.82 ± 32.46^a^	672.52 ± 27.65^a^	551.22 ± 20.54^b^	570.77 ± 51.98^b^
250–53 μm	610.73 ± 69.02^bc^	651.56 ± 63.29^bc^	1204.28 ± 27.93^a^	569.14 ± 32.19^c^	700.58 ± 101.17^b^
LF	33.25 ± 2.82^b^	38.91 ± 5.08^b^	49.29 ± 1.96^a^	32.55 ± 2.19^b^	48.20 ± 1.23^a^
iPOM	494.02 ± 18.08^bc^	490.93 ± 25.42^bc^	1085.11 ± 144.48^a^	430.28 ± 22.54^c^	589.88 ± 26.90^b^
mSOM	50.19 ± 1.68^b^	47.77 ± 6.81^bc^	66.96 ± 1.77^a^	69.75 ± 9.61^a^	44.12 ± 6.95^c^
<53 μm	320.56 ± 22.59^b^	292.77 ± 12.43^b^	640.24 ± 74.48^a^	345.13 ± 24.54^b^	318.21 ± 19.66^b^

Data are expressed as mean ± SE, n = 3. Different letters indicate statistical significance at *P* < 0.05 among fertilization treatments. *Abbreviations*: LF, Light fraction; iPOM, intra-aggregate POM; mSOM, mineral associated SOM; The abbreviations for fertilization treatments are the same as presented in Table 1.

**Table 3:** The *δ* ^13^C values of soil fractions (0–20 cm) under long-term fertilization.

Fractions	N	NPK	*δ*^13^C (‰)	SNPK	CK
			MNPK		
>2000 μm	−20.01 ± 0.27^b^	−20.43 ± 0.68^b^	−20.16 ± 0.09^b^	−19.11 ± 0.32^a^	−21.47 ± 0.49^c^
LF	−21.84 ± 0.78^bc^	−20.72 ± 1.77^ab^	−21.72 ± 0.66^bc^	−19.41 ± 0.54^a^	−22.67 ± 0.59^c^
iPOM	−20.07 ± 0.12^b^	−20.27 ± 0.47^b^	−19.94 ± 0.14^b^	−19.22 ± 0.49^a^	−21.20 ± 0.22^c^
mSOM	−17.51 ± 0.51^cd^	−16.01 ± 0.87^ab^	−16.38 ± 0.98^bc^	−14.97 ± 0.84^a^	−18.17 ± 0.52^d^
2000–250 μm	−20.21 ± 0.38^ab^	−20.69 ± 0.01^ab^	−20.26 ± 0.09^ab^	−19.28 ± 0.29^a^	−21.04 ± 1.80^b^
LF	−21.72 ± 0.77^a^	−21.96 ± 0.90^a^	−21.69 ± 0.92^a^	−21.31 ± 1.48^a^	−22.94 ± 0.19^a^
iPOM	−19.50 ± 0.93^ab^	−20.17 ± 0.12^b^	−19.69 ± 0.28^ab^	−19.15 ± 0.53^a^	−21.30 ± 0.43^c^
mSOM	−16.85 ± 0.38^c^	−15.98 ± 0.04^ab^	−16.38 ± 0.81^bc^	−15.36 ± 0.37^a^	−20.74 ± 0.22^d^
250–53 μm	−19.16 ± 0.07^c^	−18.59 ± 0.03^bc^	−17.74 ± 0.19^b^	−14.61 ± 1.49^a^	−20.71 ± 0.61^d^
LF	−21.36 ± 0.55^a^	−21.93 ± 0.08^ab^	−21.20 ± 0.05^a^	−21.68 ± 0.78^ab^	−22.24 ± 0.22^b^
iPOM	−20.61 ± 0.19^b^	−20.52 ± 0.49^b^	−20.26 ± 0.15^ab^	−19.72 ± 0.10^a^	−21.48 ± 0.58^c^
mSOM	−20.03 ± 0.67^a^	−21.11 ± 0.38^bc^	−20.64 ± 0.32^ab^	−20.34 ± 0.37^ab^	−21.82 ± 0.55^c^
<53 μm	−20.34 ± 0.38^ab^	−20.86 ± 0.15^bc^	−20.19 ± 0.27^a^	−19.84 ± 0.18^a^	−21.19 ± 0.56^c^

Data are expressed as mean ± SE, n = 3. Different letters indicate statistical significance at *P* < 0.05 among fertilization treatments. *Abbreviations*: LF, Light fraction; iPOM, intra-aggregate POM; mSOM, mineral associated SOM; The abbreviations for fertilization treatments are the same as presented in Table 1.

**Table 4:** New C input (*f*_*new*_) and decay rate (*k*, yr^−1^) of soil C in aggregate size classes and density fractions (0–20 cm) under long-term fertilization.

Fractions	*f*_*new*_ (%)				Decay rate (*k*) of old C			
	N	NPK	MNPK	SNPK	N	NPK	MNPK	SNPK
>2000 μm	19.84 ± 3.66^b^	13.55 ± 2.81^b^	21.16 ± 1.43^ab^	30.49 ± 4.09^a^	0.009 ± 0.002^b^	0.006 ± 0.001^b^	0.010 ± 0.001^b^	0.015 ± 0.002^a^
LF	9.66 ± 1.58^b^	21.90 ± 2.98^ab^	12.85 ± 2.03^b^	36.33 ± 5.09^a^	0.004 ± 0.001^b^	0.011 ± 0.002^ab^	0.006 ± 0.002^b^	0.018 ± 0.004^a^
iPOM	15.89 ± 1.75^b^	12.61 ± 3.42^b^	21.45 ± 2.44^ab^	26.45 ± 6.52^a^	0.007 ± 0.001^b^	0.005 ± 0.001^b^	0.010 ± 0.001^ab^	0.012 ± 0.003^a^
mSOM	16.09 ± 2.60^b^	49.33 ± 9.79^ab^	61.99 ± 14.07^a^	71.76 ± 18.87^a^	0.007 ± 0.002^b^	0.029 ± 0.005^ab^	0.058 ± 0.012^a^	0.063 ± 0.011^a^
2000–250 μm	11.94 ± 2.53^b^	4.88 ± 0.20^c^	13.58 ± 1.65^b^	23.99 ± 3.97^a^	0.005 ± 0.001^b^	0.002 ± 0.000^c^	0.006 ± 0.001^b^	0.011 ± 0.002^a^
LF	13.81 ± 2.69^a^	10.73 ± 2.84^a^	16.27 ± 3.06^a^	17.68 ± 3.13^a^	0.006 ± 0.002^a^	0.004 ± 0.001^a^	0.007 ± 0.002^a^	0.008 ± 0.002^a^
iPOM	24.99 ± 2.97^a^	15.08 ± 1.56^a^	26.78 ± 4.74^a^	28.38 ± 6.94^a^	0.012 ± 0.003^a^	0.007 ± 0.001^a^	0.013 ± 0.002^a^	0.013 ± 0.002^a^
mSOM	58.50 ± 5.70^b^	68.39 ± 0.62^ab^	80.05 ± 14.91^a^	76.64 ± 5.23^a^	0.035 ± 0.006^b^	0.046 ± 0.001^ab^	0.074 ± 0.016^a^	0.059 ± 0.009^ab^
250–53 μm	23.51 ± 0.99^c^	30.61 ± 0.47^c^	54.74 ± 3.47^b^	83.35 ± 16.23^a^	0.011 ± 0.000^b^	0.015 ± 0.000^b^	0.032 ± 0.003^b^	0.087 ± 0.025^a^
LF	10.81 ± 1.81^a^	3.77 ± 0.96^b^	14.98 ± 0.69^a^	5.82 ± 1.50^b^	0.005 ± 0.001^a^	0.002 ± 0.000^b^	0.006 ± 0.000^a^	0.002 ± 0.000^b^
iPOM	11.81 ± 2.62^b^	12.49 ± 2.38^b^	19.67 ± 2.43^a^	22.66 ± 1.25^a^	0.005 ± 0.001^b^	0.005 ± 0.001^b^	0.009 ± 0.001^a^	0.010 ± 0.000^a^
mSOM	23.12 ± 4.65^a^	8.79 ± 1.79^b^	18.14 ± 3.93^ab^	18.23 ± 2.55^ab^	0.011 ± 0.002^a^	0.004 ± 0.001^b^	0.008 ± 0.002^ab^	0.008 ± 0.002^ab^
<53 μm	11.90 ± 2.44^a^	4.39 ± 0.04^b^	16.89 ± 3.63^a^	18.02 ± 2.45^a^	0.005 ± 0.001^ab^	0.002 ± 0.000^b^	0.007 ± 0.002^a^	0.008 ± 0.001^a^

Data are expressed as mean ± SE, n = 3. Different letters indicate statistical significance at *P* < 0.05 among fertilization treatments. *Abbreviations*: LF, Light fraction; iPOM, intra-aggregate POM; mSOM, mineral associated SOM; The abbreviations for fertilization treatments are the same as presented in Table 1.
